# Synthetic Formatotrophs for One‐Carbon Biorefinery

**DOI:** 10.1002/advs.202100199

**Published:** 2021-05-03

**Authors:** Junho Bang, Jung Ho Ahn, Jong An Lee, Chang Hun Hwang, Gi Bae Kim, Jinwon Lee, Sang Yup Lee

**Affiliations:** ^1^ Metabolic and Biomolecular Engineering National Research Laboratory Department of Chemical and Biomolecular Engineering (BK21 Plus Program) Institute for the BioCentury Korea Advanced Institute of Science and Technology (KAIST) Daejeon 34141 Republic of Korea; ^2^ Systems Metabolic Engineering and Systems Healthcare Cross‐Generation Collaborative Laboratory KAIST Daejeon 34141 Republic of Korea; ^3^ Department of Chemical and Biomolecular Engineering Sogang University Seoul 04107 Republic of Korea; ^4^ C1 Gas Refinery R&D Center Sogang University Seoul 04107 Republic of Korea; ^5^ BioInformatics Research Center and BioProcess Engineering Research Center KAIST Daejeon 34141 Republic of Korea

**Keywords:** formatotroph, formic acid assimilation, one‐carbon biorefinery, systems metabolic engineering

## Abstract

The use of CO_2_ as a carbon source in biorefinery is of great interest, but the low solubility of CO_2_ in water and the lack of efficient CO_2_ assimilation pathways are challenges to overcome. Formic acid (FA), which can be easily produced from CO_2_ and more conveniently stored and transported than CO_2_, is an attractive CO_2_‐equivalent carbon source as it can be assimilated more efficiently than CO_2_ by microorganisms and also provides reducing power. Although there are native formatotrophs, they grow slowly and are difficult to metabolically engineer due to the lack of genetic manipulation tools. Thus, much effort is exerted to develop efficient FA assimilation pathways and synthetic microorganisms capable of growing solely on FA (and CO_2_). Several innovative strategies are suggested to develop synthetic formatotrophs through rational metabolic engineering involving new enzymes and reconstructed FA assimilation pathways, and/or adaptive laboratory evolution (ALE). In this paper, recent advances in development of synthetic formatotrophs are reviewed, focusing on biological FA and CO_2_ utilization pathways, enzymes involved and newly developed, and metabolic engineering and ALE strategies employed. Also, future challenges in cultivating formatotrophs to higher cell densities and producing chemicals from FA and CO_2_ are discussed.

## Graphical abstract

This paper reviews the recent advances in developing synthetic FA and CO_2_ assimilation pathways and formatotrophs. Moreover, chemicals that can be produced from FA and CO_2_ together with the strategies for metabolic engineering of the formatotrophic *Escherichia coli* to produce these chemicals are discussed. This review provides things to consider for building a sustainable one‐carbon biorefinery.

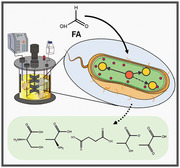

## 1 Introduction

Bio‐based production of chemicals, fuels, and materials has been mostly relying on carbohydrates prepared from biomass as raw materials. The use of CO_2_ as a carbon source for microbial production of chemicals^[^ ^1^, ^2^, ^3^, ^4^ ^]^ has attracted much attention as CO_2_ is abundant (e.g., industrial off‐gas) and inexpensive.^[^ ^5^, ^6^ ^]^ Moreover, the utilization of CO_2_ as a carbon source can contribute to reducing atmospheric CO_2_, which is beneficial to cope with climate crisis.^[^ ^7^, ^8^ ^]^ Hence, numerous studies have been carried out to increase CO_2_ assimilation using natural CO_2_ assimilation pathways,^[^ ^9^, ^10^, ^11^, ^12^ ^]^ including the Calvin–Benson–Bassham (CBB) cycle, and to use these pathways for the conversion of CO_2_ to chemicals.^[^ ^13^, ^14^, ^15^, ^16^ ^]^ However, the carboxylases, such as ribulose‐1,5‐bisphosphate carboxylase/oxygenase (Rubisco), involved in the natural CO_2_ assimilation pathways exhibit low catalytic rates and have a tendency to confuse CO_2_ with O_2_.^[^ ^17^ ^]^ In addition, engineering of Rubisco for more efficient carboxylation has not been successful due to the existence of trade‐off between the maximum turnover rate and CO_2_/O_2_ affinity.^[^ ^17^, ^18^ ^]^ The reason for such behavior is unclear as the reaction mechanism of this enzyme has not been fully studied.

To overcome the limitation of direct CO_2_ assimilation in the natural CO_2_ assimilation pathways, formic acid (FA) and methanol, both of which can be produced by electrochemical reduction of CO_2_,^[^ ^19^, ^20^ ^]^ have been employed as alternative carbon sources equivalent to CO_2_ for the following reasons. FA and methanol are easier to store and transport than CO_2_;^[^ ^21^, ^22^, ^23^, ^24^ ^]^ FA and methanol can be assimilated by microorganisms more efficiently than CO_2_ as both chemicals are more soluble in water and can be metabolized better compared with CO_2_.^[^ ^22^ ^]^ Between FA and methanol, FA is a better carbon source than methanol as pure FA can be easily produced from CO_2_ using electrochemical^[^ ^19^ ^]^ or catalytic processes,^[^ ^25^ ^]^ while the production of methanol from CO_2_ is less efficient as CO and FA are also formed as byproducts.^[^ ^26^ ^]^ Thus, this review focuses on the studies associated with the utilization of FA as a carbon source together with CO_2_.

Native formatotrophs, such as *Methylobacterium extorquens*, can grow using FA as a sole carbon source.^[^ ^27^ ^]^ However, native formatotrophs grow slowly and are sensitive to culture conditions. Also, they are rather difficult to metabolically engineer due to the lack of efficient genetic manipulation tools compared with well‐known host strains such as *Escherichia coli*. In addition, the native FA assimilation pathways, including serine and reductive acetyl‐CoA pathways, present in the native formatotrophs are kinetically and energetically inefficient.^[^ ^23^ ^]^ Thus, studies on FA assimilation have mainly been carried out using metabolically engineered microorganisms (e.g., *E. coli*) harboring synthetic FA assimilation pathways.^[^ ^28^, ^29^, ^30^, ^31^, ^32^, ^33^, ^34^, ^35^ ^]^ Taking advantage of well‐established methods and strategies for metabolic engineering, *E. coli* has often been employed as a base strain to establish synthetic FA assimilation pathways and develop synthetic formatotrophs.

In recent studies, various synthetic formatotrophic *E. coli* strains have been developed to establish one‐carbon biorefinery.^[^ ^33^, ^34^, ^35^ ^]^ However, the highest cell density achieved by the formatotrophic *E. coli* strain was 3.47 g dry cell weight (gDCW) L^−1^, which is significantly lower than that (128 gDCW L^−1^) of an *E. coli* strain cultivated using a conventional carbon source such as glucose.^[^ ^36^ ^]^ The main reasons for the poor cell growth of formatotrophic *E. coli* strain are: low FA assimilation efficiency,^[^ ^31^ ^]^ insufficient generation of reducing power from FA,^[^ ^34^ ^]^ and low FA tolerance of *E. coli*.^[^ ^35^, ^37^, ^38^ ^]^ Thus, these problems need to be solved to establish an economically competitive one‐carbon biorefinery.

In this paper, we review recent advances in constructing FA assimilation pathways and developing formatotrophic *E. coli* strains. Also, metabolic engineering strategies for further improving the growth of the formatotrophic *E. coli* strains are discussed. In addition, we present strategies for the metabolic engineering of formatotrophic *E. coli* to produce several example chemicals, including lactic acid (LA), L‐alanine, L‐serine, and succinic acid (SA), from FA and CO_2_ as sole carbon sources. Finally, fermentation processes that can further enhance formatotrophic growth of *E. coli* and production of chemicals from FA and CO_2_ are suggested.

## 2 Overview on the Development of Formatotrophic *E. coli* Strains

**Figure 1:**
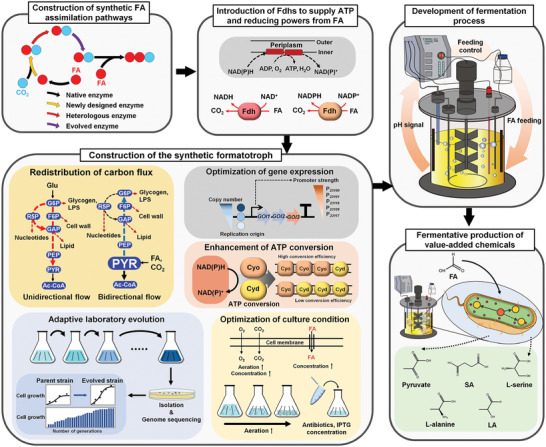
Overall strategies of systems metabolic engineering for the bio‐based production of chemicals from FA and CO_2_. Synthetic formatotrophs are developed by constructing synthetic FA assimilation pathways, introducing Fdhs to supply ATP and reducing powers from FA, and employing ALE and/or rational metabolic engineering strategies, such as redistribution of carbon flux, optimization of gene expression, enhancement of ATP conversion, and optimization of culture condition. Then, fermentation processes are developed to facilitate enhanced formatotrophic growth. Finally, the synthetic formatotrophs are metabolically engineered for fermentative production of chemicals. Abbreviations are: Ac‐CoA, acetyl‐CoA; Cyd, cytochrome bd‐I ubiquinol oxidase; Cyo, cytochrome bo3 ubiquinol oxidase; F6P, fructose 6‐phosphate; FA, formic acid; G6P, glucose 6‐phosphate; GAP, glyceraldehyde 3‐phosphate; Glu, glucose; GOI, gene of interest; LPS, lipopolysaccharide; PEP, phosphoenolpyruvate; PYR, pyruvate; R5P, ribose 5‐phosphate; SA, succinic acid; LA, lactate; Fdh, formate dehydrogenase.

**Figure 2:**
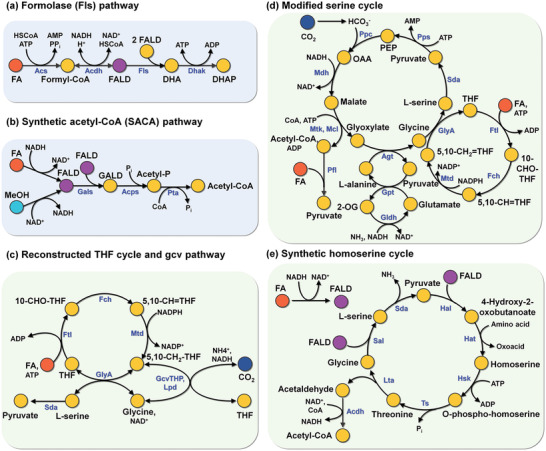
Synthetic pathways for FA or FA and CO_2_ assimilations. a) Fls pathway, b) SACA pathway, c) rTHF‐rgcv pathway, d) modified serine cycle, and e) synthetic homoserine cycle. All of the synthetic FA assimilation pathways reported to date are shown. Pathways in blue‐colored boxes are developed using de novo enzymes. Pathways in green‐colored boxes are developed by reconstruction of FA assimilation pathways. Enzymes involved in the synthetic pathways are indicated in blue. Abbreviations are: 2‐OG, 2‐oxoglutarate; 5,10‐CH_2_—THF, 5,10‐methylenetetrahydrofolate; 5,10‐CH=THF, 5,10‐methenyltetrahydrofolate; 10‐CHO—THF, 10‐formyltetrahydrofolate; Acdh, acetaldehyde dehydrogenase; Acetyl‐P, acetyl‐phosphate; Acps, acetyl‐phosphate synthase; Acs, acetyl‐CoA synthase; Agt, alanine‐glyoxylate transaminase; Dhak, dihydroxyacetone kinase; FA, formic acid; FALD, formaldehyde; DHA, dihydroxyacetone; DHAP, dihydroxyacetone‐phosphate; Fch, 5,10‐CH=THF cyclohydrolase; Fls, formolase; Ftl, formate‐tetrahydrofolate ligase; GALD, glycolaldehyde; Gals, glycolaldehyde synthase; GcvTHP, gcv complex; Gldh, glutamate dehydrogenase; GlyA, serine hydroxymethyltransferase; Gpt, glutamate‐pyruvate transaminase; Hal, 4‐hydroxy‐2‐oxobutanoate aldolase; Hat, 4‐hydroxy‐2‐oxobutanoate aminotransferase; Hsk, homoserine kinase; Lpd, lipoamide dehydrogenase; Lta, threonine aldolase; Mcl, malyl‐CoA lyase; Mdh, malate dehydrogenase; MeOH, methanol; Mtd, 5,10‐CH_2_—THF dehydrogenase; Mtk, malate thiokinase; OAA, oxaloacetate; PEP, phosphoenolpyruvate; Pfl, pyruvate formate lyase; Ppc, phosphoenolpyruvate carboxylase; Pps, phosphoenolpyruvate synthase; Pta, phosphate acetyltransferase; Sal, serine aldolase; Sda, serine deaminase; THF, tetrahydrofolate; Ts, threonine synthase.

Studies on the development of formatotrophic *E. coli* strains up to date can be summarized as follows. To enable *E. coli* to utilize FA only or FA and CO_2_, various synthetic FA assimilation pathways (**Table** **1**) were constructed by the development of novel enzymes,^[^ ^39^ ^]^ enzyme engineering,^[^ ^40^ ^]^ and reconstruction of FA assimilation pathways (**Figure** **1**).^[^ ^28^, ^29^, ^30^, ^31^ ^]^ The synthetic FA assimilation pathways lead to the biosynthesis of one of the three core metabolites, acetyl‐CoA, dihydroxyacetone phosphate (DHAP), and pyruvate, from FA only or FA and CO_2_ (**Figure** **2**). In these initial studies, however, other carbon sources such as glucose were still needed to provide reducing power for cell growth. Thus, strategies for generating reducing power from FA by introducing a heterologous formate dehydrogenase (Fdh) was employed (Figure 1).^[^ ^31^ ^]^ Furthermore, adaptive laboratory evolution (ALE) and/or rational metabolic engineering were performed using the FA and CO_2_ assimilating *E. coli* strains to achieve formatotrophic growth (Figure 1 and **Table** **2**).^[^ ^33^, ^34^, ^35^ ^]^ In a previous study, the Rubisco‐dependent heterotrophic *E. coli* strain was evolved to achieve formatotrophic growth up to an optical density (*OD*_600_) of 0.28 by cultivating the cells in a xylose‐limited chemostat with excess sodium FA and continuous sparging of CO_2_‐enriched air.^[^ ^33^ ^]^ In another study, a short‐term ALE by serial cultivations of a formatotorophic *E. coli* strain, which was developed by introducing a synthetic FA and CO_2_ assimilation pathway in the serine‐auxotrophic *E. coli* strain, improved formatotrophic growth enabling the formatotorophic *E. coli* strain to grow up to an *OD*_600_ of 1.0.^[^ ^34^ ^]^ In a most recent study, a rationally engineered *E. coli* strain capable of growing to a relatively high cell density (*OD*_600_ of ≈11) using FA and CO_2_ as sole carbon sources^[^ ^35^ ^]^ was developed. We describe detailed strategies employed for developing these formatotrophic *E. coli* strains below.

**Table 1:** Summary of the synthetic FA or FA and CO_2_ assimilation pathways

Pathway	Substrate(s)	Product	Required amount of ATP and reducing power^a)^	Description	Reference(s)
Formolase pathway	FA	Dihydroxyacetone phosphate	4 ATP and 3 NADH	Employs de novo enzyme (Fls) FA assimilation was only demonstrated in vitro	^[^ ^39^ ^]^
Synthetic acetyl‐CoA pathway	FA	Acetyl‐CoA	2 ATP and 2 NADH	Employs de novo enzyme (Gals) FA assimilation was demonstrated in vivo	^[^ ^40^ ^]^
Reconstructed THF cycle and reverse glycine cleavage pathway	FA and CO_2_	Pyruvate	2 ATP, 1 NADH, and 2 NADPH	THF cycle was reconstructed by employing heterologous enzymes	^[^ ^29^, ^30^, ^31^ ^]^
Modified serine cycle	FA and CO_2_	Acetyl‐CoA	3 ATP, 1 NADH, and 2 NADPH	Serine cycle was reconstructed by employing heterologous enzymes Consists longer metabolic pathway compared with other synthetic FA assimilation pathways	^[^ ^47^ ^]^
Synthetic homoserine cycle	FA	Acetyl‐CoA	1 ATP and 1 NADH	Less energy required compared with the modified serine cycle Shorter metabolic pathway compared with the modified serine cycle	^[^ ^48^ ^]^

^a)^ Total amount of ATP and reducing powers (NADH and NADPH) required to synthesize one molecule of product from FA or FA and CO_2_.

**Table 2:** Growth performances of various formatotrophic *E. coli* strains using FA and CO_2_ as sole carbon sources

Strain	Initial *OD*_600_	Final *OD*_600_	Cultivation time [h]	Doubling time [h]	FA assimilation pathway^b)^	Description	Reference
*E. coli*	0.91	11.1	577	157.6	rTHF‐rgcv and Fdh	Constructed by rational metabolic engineering Highest maximum cell growth solely on FA and CO_2_	^[^ ^35^ ^]^
*E. coli*	0.31	0.5	77	N/A^c)^	rTHF‐rgcv and Fdh	Constructed by rational metabolic engineering First demonstration of the formatotrophic growth in *E. coli*	^[^ ^31^ ^]^
*E. coli*	0.01^a)^	0.28^a)^	120^a)^	18 ± 4	CBB and Fdh	Constructed by ALE Carbons are provided by CO_2_ while FA is oxidized to produce energy source	^[^ ^33^ ^]^
*E. coli*	0.01^a)^	1.0^a)^	60	7.7	rTHF‐rgcv and Fdh	Constructed by ALE Fastest cell growth solely on FA and CO_2_	^[^ ^34^ ^]^

Abbreviations are rTHF, reconstructed tetrahydrofolate cycle; rgcv, reverse glycine cleavage pathway; Fdh, formate dehydrogenase; CBB, Calvin–Benson–Bassham cycle; ALE, adaptive laboratory evolution.

^a)^ Values estimated using the data provided in corresponding study.

^b)^ FA assimilation pathway employed by the formatotrophic *E. coli* strain.

^c)^ Not available.

## 3 Formic Acid Assimilation to Intracellular Metabolites through Synthetic Formic Acid Assimilation Pathways

### 3.1 Development of Synthetic Formic Acid Assimilation Pathways

To produce chemicals from FA in *E. coli*, FA needs to be assimilated to intracellular metabolites. For this purpose, synthetic FA assimilation pathways were constructed using de novo enzymes catalyzing the FA assimilation reactions and/or by reconstructing the FA assimilation pathways.

In a previous study, the synthetic formolase (Fls) pathway^[^ ^39^ ^]^ was employed in *E. coli* to produce one DHAP molecule from three FA molecules using four ATP and three NADH molecules (Figure 2a and Table 1). In this pathway, FA is converted to DHAP through four steps of enzymatic reactions. First, FA is converted to formyl‐CoA by native acetyl‐CoA synthase (Acs) and further converted to formaldehyde (FALD) by *Listeria monocytogenes* acetaldehyde dehydrogenase (Acdh). Next, one molecule of dihydroxyacetone (DHA) is synthesized through carbon‐carbon bonding of three molecules of FALD by Fls, which is an enzyme developed in this study. The Fls was designed by computational method using *Pseudomonas fluorescens* benzaldehyde lyase as a template. The binding pocket of benzaldehyde lyase for benzaldehyde was engineered to possess higher affinity toward FALD based on RosettaDesign^[^ ^43^ ^]^ and Foldit^[^ ^44^ ^]^ and the catalytic efficiency of Fls was improved by further performing error‐prone PCR. Finally, DHA kinase (Dhak) was utilized to convert DHA to DHAP, which is further utilized to synthesize intracellular metabolites through glycolysis and gluconeogenesis. The Fls pathway can be easily introduced into other microorganisms, since only four different enzymes are required to operate this pathway. However, low enzyme activity of the Fls was a major drawback for FA assimilation through the Fls pathway. Due to low enzyme activity of the Fls (catalytic efficiency; *kcat*/*km* = 4.7 s^−1^ M^−1^), FA assimilation through the Fls pathway was only confirmed in vitro. Thus, employing the Fls pathway in *E. coli* for FA assimilation can be challenging.

In another study, the synthetic acetyl‐CoA (SACA) pathway^[^ ^40^ ^]^ was introduced in *E. coli* to produce one acetyl‐CoA molecule from two FA molecules using two ATP and two NADH molecules (Figure 2b and Table 1). In this pathway, FALD is first synthesized from FA by Acs and Acdh, similar to the Fls pathway.^[^ ^39^ ^]^ Next, one molecule of glycolaldehyde is synthesized from two FALD molecules by glycolaldehyde synthase (Gals), which is an enzyme developed in this study. The Gals was developed by directed evolution of *Pseudomonas putida* benzoylformate decarboxylase (Bfd). Since Fls was demonstrated to synthesize DHA from FALD,^[^ ^39^ ^]^ the amino acid sequences of Bfd and Fls were aligned to predict the FALD binding site in the Bfd and the residues of the Bfd, which correspond to the key residues in the FALD binding site of the Fls, were mutated to develop Gals. Finally, *Saccharomonospora marina* acetyl‐phosphate synthase was employed to convert glycolaldehyde to acetyl‐phosphate, which is further converted to acetyl‐CoA by native phosphate acetyltransferase (Pta). The synthetic SACA pathway, which includes five reaction steps, enables acetyl‐CoA production from FALD. However, employing the SACA pathway in *E. coli* for FA assimilation can be challenging due to the low enzyme activity of Gals (*kcat*/*km* = 9.29 s^−1^ M^−1^). It should be noted that the catalytic efficiency of *Moorella thermoacetica* formate‐tetrahydrofolate (THF) ligase (Ftl), which is a natural FA assimilating enzyme, is 2450.98 s^−1^ M^−1^.^[^ ^45^ ^]^

The utilization of de novo enzymes allows construction of short and simple synthetic FA assimilation pathways and production of target chemicals directly from FA. However, low enzyme activities of the novel enzymes are a major drawback for the synthetic FA assimilation pathways employing such enzymes. On the other hand, the native FA assimilation pathways consist of more enzymatic reactions than the synthetic FA assimilation pathways employing de novo enzymes. In addition, it is difficult to employ the native FA assimilation pathways for chemical production as the reactions involved in the native FA assimilation pathways are kinetically (e.g., Rubisco)^[^ ^17^ ^]^ and energetically inefficient (e.g., CBB cylce requires 7 ATP and 4 NAD(P)H to synthesize 1 acetyl‐CoA molecule; refer to Table 1 for comparison).^[^ ^23^ ^]^ Also, the pathway produces toxic intermidiates, such as hydroxypyruvate.^[^ ^46^ ^]^ Thus, a different approach that involves the reconstruction of FA assimilation pathways was undertaken to develop synthetic FA assimilation pathways.

In previous studies, the synthetic FA assimilation pathways comprising the reconstructed THF cycle and reverse glycine cleavage pathway (Figure 2c and Table 1), named as rTHF‐rgcv pathway hereafter, were constructed in *E. coli* to enable FA assimilation.^[^ ^29^, ^30^, ^31^ ^]^ In these pathways, one molecule of FA is incorporated into THF by the heterologous expression of the *ftl* gene (encoding Ftl), producing formyl‐THF (10‐CHO—THF). Next, the heterologous 5,10‐methenyl‐THF (5,10‐CH=THF) cyclohydrolase (Fch) converts 10‐CHO—THF to 5,10‐CH=THF, which is further converted to 5,10‐methylene‐THF (5,10‐CH_2_—THF) by the heterologous 5,10‐CH_2_—THF dehydrogenase (Mtd). The heterologous genes encoding Ftl, Fch, and Mtd were all obtained from *Clostridium ljungdahlii* ^[^ ^29^ ^]^ or *M. extorquens*.^[^ ^30^, ^31^ ^]^ Then, 5,10‐CH_2_—THF and glycine, which is synthesized using 5,10‐CH_2_—THF, CO_2_, and NH_3_ through the gcv pathway, were converted to THF and L‐serine by the native serine hydroxymethyltransferase (GlyA). Finally, pyruvate is produced from L‐serine by the native serine deaminase (Sda). The synthetic FA assimilation pathway, comprising rTHF‐rgcv pathway, synthesizes one pyruvate molecule from two FA molecules and one CO_2_ molecule, consuming two ATP, two NADPH, and one NADH molecules (Table 1). This synthetic FA assimilation pathway is promising because the enzymes employed for FA assimilation were selected from native formatotrophs, which possess much higher enzyme activities^[^ ^45^ ^]^ than those of de novo enzymes.^[^ ^39^, ^40^ ^]^ In addition, the theoretical maximum biomass yield by this synthetic FA assimilation pathway (5.7 gDCW mol of FA^−1^) was calculated to be the highest compared to those of other native and synthetic FA assimilation pathways (CBB cycle, 4.8 gDCW mol of FA^−1^; Fls pathway, 4.9 gDCW mol of FA^−1^; serine cycle, 5 gDCW mol of FA^−1^) based on flux balance analysis using *E. coli* metabolic model.^[^ ^41^ ^]^ Most importantly, pyruvate can be easily converted to diverse chemicals through the native metabolic pathways in *E. coli*.

In another study, a modified serine cycle, which produces one pyruvate molecule from two FA and one CO_2_ molecules, was constructed in *E. coli* (Figure 2d and Table 1).^[^ ^47^ ^]^ In this pathway, FA and glycine are used to produce L‐serine through the THF cycle and the synthesized L‐serine is further utilized to run the serine cycle. In the serine cycle, L‐serine is converted to phosphoenolpyruvate (PEP) by sequential reactions of Sda and PEP synthase (encoded by the *ppsA* gene). Next, CO_2_ is assimilated by the native PEP carboxylase to convert PEP to oxaloacetate, which is further converted to malate by the native malate dehydrogenase. The synthesized malate is converted to malyl‐CoA by the native malate thiokinase and further broken down into glyoxylate and acetyl‐CoA by malyl‐CoA lyase. Finally, acetyl‐CoA is converted to pyruvate by the reverse reaction of native pyruvate formate lyase (Pfl) and glyoxylate is converted to glycine, which is reutilized to operate the THF cycle, by the native alanine‐glyoxylate transaminase.^[^ ^42^ ^]^ Although the modified serine cycle produces pyruvate from FA and CO_2_, similar to the synthetic FA assimilation pathway comprising the rTHF‐rgcv pathway, it displays two weaknesses. First, the modified serine cycle requires three ATP, one NADPH, and two NADH molecules (Table 1). Thus, one more ATP is consumed to operate the modified serine cycle for the production of one pyruvate molecule than operating the synthetic FA assimilation pathway comprising the rTHF‐rgcv pathway (Table 1). Second, the modified serine cycle is complex and employs more enzymatic reactions than the synthetic FA assimilation pathway comprising the rTHF‐rgcv pathway, which is an undesirable feature for further metabolic engineering.

In a most recent study, the synthetic homoserine cycle was constructed in *E. coli* by the reconstruction of serine cycle (Figure 2e and Table 1).^[^ ^48^ ^]^ In this pathway, FALD, which is produced from FA by Acs and Acdh, is incorporated into glycine to produce L‐serine by the native serine aldolase. L‐serine is deaminated by the native Sda to produce pyruvate and an additional FALD molecule is combined with pyruvate to synthesize 4‐hydroxy‐2‐oxobutanoate (HOB) using native 4‐hydroxy‐2‐oxobutanoate aldolase. Next, the native HOB aminotransferase converts HOB to homoserine, which is further converted to glycine and acetaldehyde by sequential reactions of native homoserine kinase, threonine synthase, and threonine aldolase. Finally, glycine is reused for the production of L‐serine while acetaldehyde is converted to acetyl‐CoA by the native Acdh. The synthetic homoserine cycle synthesizes one acetyl‐CoA molecule from two FA molecules consuming one ATP and one NADH molecules, while the modified serine cycle consumes three ATP, one NADPH, and two NADH molecules to synthesize one acetyl‐CoA molecule (Figure 2d and Table 1). Thus, the synthetic homoserine cycle is more energy efficient than the modified serine cycle. However, FALD, which is a cytotoxic compound,^[^ ^49^ ^]^ is produced as an intermediate in the synthetic homoserine cycle during FA assimilation. Moreover, biosynthesis of acetyl‐CoA from FA is less desirable than production of pyruvate or DHAP.

Among the synthetic FA assimilation pathways developed to date, the rTHF‐rgcv pathway is the best choice for FA and CO_2_ assimilation. The rTHF‐rgcv pathway is simple and requires fewer enzymatic reactions than the modified serine cycle. In addition, while the rTHF‐rgcv pathway directly assimilates FA and CO_2_, the Fls pathway, SACA pathway, and synthetic homoserine cycle require an additional step for converting FA to FALD, which is highly toxic to living cells.^[^ ^49^ ^]^ Therefore, FA assimilation in *E. coli* using the rTHF‐rgcv pathway will be the focus for the remainder of this review.

### 3.2 Further Engineering of the Reconstructed rTHF‐rgcv Pathway

**Figure 3:**
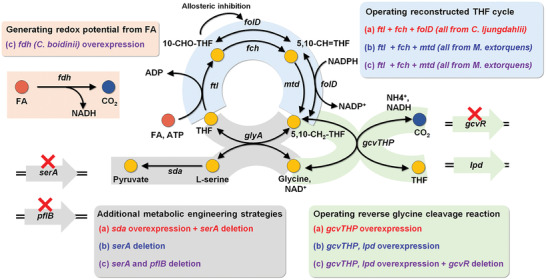
Strategies to enhance FA and CO_2_ assimilation efficiency of the rTHF‐rgcv pathway. Metabolic engineering strategies employed in three recent studies (a) Tashiro et al.;^[^ ^29^ ^]^ b) Yishai et al.;^[^ ^30^ ^]^ c) Bang and Lee^[^ ^31^ ^]^) to enhance FA and CO_2_ assimilation efficiency of the rTHF‐rgcv pathway are shown. Similar engineering strategies employed in three recent studies are grouped together in colored boxes. Red X marks represent gene deletion. Abbreviations are: 5‐10‐CH=THF, 5,10‐methenyl THF; 5,10‐CH_2_—THF, 5,10‐methlylene THF; 10‐CHO—THF, 10‐formyl THF; FA, formic acid; *fch*, 5,10‐CH=THF cyclohydrolase; *fdh*, formate dehydrogenase; *folD*, bifunctional 5,10‐CH_2_—THF dehydrogenase/5,10‐CH_2_—THF cyclohydrolase; *ftl*, formate‐tetrahydrofolate ligase; *gcvR*, transcriptional regulator of glycine cleavage complex; *gcvTHP*, gcv complex; *glyA*, serine hydroxymethyltransferase; *lpd*, lipoamide dehydrogenase; *mtd*, 5,10‐CH_2_—THF dehydrogenase; *sda*, serine deaminase; *serA*, phosphoglycerate dehydrogenase; THF, tetrahydrofolate.

To improve FA assimilation to intracellular metabolites using the rTHF‐rgcv pathway, the native metabolic pathways in *E. coli* had to be engineered because the native metabolic pathways could influence the FA assimilation efficiency of the rTHF‐rgcv pathway. In a study by Tashiro et al.,^[^ ^29^ ^]^ the FA assimilation efficiency of the *E. coli* strain equipped with the rTHF‐rgcv pathway was enhanced by the introduction of the *ftl* and *fch* genes from *C. ljungdahlii* and the replacement of native *folD* gene encoding the bifunctional 5,10‐CH_2_—THF dehydrogenase/5,10‐CH_2_—THF cyclohydrolase, which is allosterically inhibited by 10‐CHO—THF,^[^ ^50^ ^]^ with the *C. ljungdahlii folD* gene (**Figure** **3**). In addition, the native *gcvTHP* operon encoding gcv complex was overexpressed to improve glycine production from CO_2_ and 5,10‐CH_2_—THF. Moreover, the *serA* gene encoding phosphoglycerate dehydrogenase was deleted to allow L‐serine production only from FA and CO_2_ by preventing L‐serine production from glucose. Finally, the native *sda* gene was overexpressed to increase conversion of L‐serine to pyruvate. Although the metabolic engineering strategies employed in the study by Tashiro et al. had increased FA assimilation in *E. coli*, the level of FA assimilation to L‐serine and pyruvate was insufficient to facilitate formatotrophic growth; less than 10% and 1.5% of proteinogenic L‐serine and total pyruvate, respectively, were synthesized from FA and CO_2_.

FA assimilation of the engineered *E. coli* strain possessing the rTHF‐rgcv pathway was further improved by Yishai et al.^[^ ^30^ ^]^ and by Bang and Lee^[^ ^31^ ^]^ (Figure 3). Similar metabolic engineering strategies were employed in both studies to develop an *E. coli* strain capable of efficiently assimilating FA. The rTHF cycle was first established by the introduction of *M. extorquens ftl* and the replacement of native *folD* gene with the *M. extorquens fch* and *mtd* genes, as *M. extorquens* Fch and Mtd are unifunctional and not allosterically inhibited by 10‐CHO—THF.^[^ ^31^ ^]^ Next, the native *gcvTHP* operon was overexpressed to increase gcv reaction and the native *lpd* gene encoding lipoamide dehydrogenase, which supply NADH to the gcv complex, was overexpressed to enhance NADH supply. Furthermore, the native *serA* gene was deleted to ensure L‐serine is only produced from FA and CO_2_ only. Engineering of the rTHF‐rgcv pathway in the study by Yishai et al. was settled at this stage and the engineered *E. coli* strain reported in this study synthesized L‐serine from FA and CO_2_ with high efficiency; up to 90% and 10% of proteinogenic L‐serine and total pyruvate, respectively, were produced from FA and CO_2_. Further metabolic engineering strategies were employed in the study by Bang and Lee to derive a better FA assimilating *E. coli* strain. The *gcvR* gene encoding transcriptional regulator of glycine cleavage complex was deleted and the native *gcvTHP* operon was overexpressed by replacing the native promoter to a strong *trc* promoter. Next, the *pfl* gene encoding Pfl was deleted to prevent degradation of pyruvate to FA and acetyl‐CoA. Finally, the *Candida boidinii fdh* gene encoding NAD^+^ utilizing Fdh was introduced to reduce the engineered strain's dependence on glucose. The final engineered *E. coli* strain synthesized 98% and 15% of proteinogenic L‐serine and total pyruvate, respectively, from FA and CO_2_, which is better than that reported in the study by Yishai et al.^[^ ^30^ ^]^ Due to previous studies on the development of efficient synthetic FA assimilation pathways, FA can be utilized to produce chemicals in *E. coli*. However, the metabolically engineered strains’ dependencies on other carbon sources (e.g., glucose) for cell growth were still unresolved even at this stage. Thus, ALE and/or rational metabolic engineering were further carried out to develop the formatotrophic *E. coli* strain capable of growing solely from FA and CO_2_.

## 4 Development of a Synthetic Formatotroph Capable of Growing on Formic Acid and CO_2_

### 4.1 Development of a Synthetic Formatotroph Using ALE

In the case of formatotrophic *E. coli*, the FA assimilation pathway synthesizes intracellular metabolites such as nucleotides, cell wall, and lipids from FA, while Fdh regenerates NADH and NADPH using FA to provide cellular energy and reducing power required for FA assimilation and intracellular metabolite synthesis. However, the metabolically engineered *E. coli* strains equipped with the rTHF‐rgcv pathway and Fdh could not grow using FA and CO_2_ as sole carbon sources,^[^ ^35^ ^]^ indicating that the metabolic system of the engineered *E. coli* strain needs to be further modified to achieve formatotrophic growth.

Although the overall metabolism of *E. coli* is well studied, the complexity of the metabolic system makes the identification of engineering targets (i.e., metabolic pathways, enzymes, and regulatory proteins) and selection of optimal methods for engineering those targets in *E. coli* difficult. Since the ultimate objective of metabolic engineering in this case is constructing a strain capable of growing solely on FA and CO_2_, actual cultivation of all rationally engineered strains needs to be performed to examine the effectiveness of metabolic engineering. Since this is quite labor intensive, ALE can be an alternative strategy to construct a synthetic formatotroph while avoiding such difficulties (Figure 1).

In a previous study, the Rubisco‐dependent heterotrophic parental *E. coli* strain was developed by introducing the *Synechococcus elongatus* phosphoribulokinase, *Rhodospirillum rubrum* Rubisco, and *Pseudomonas sp*. 101 Fdh, while deleting the *pfkAB* and *zwf* genes encoding phosphofructokinase and 6‐phosphate‐1‐dehydrogenase, respectively.^[^ ^33^ ^]^ The parental strain, which was constructed to convert pentose sugar (i.e., xylose) to biomass precursors only through the carboxylation by Rubisco,^[^ ^51^ ^]^ was cultured in a xylose‐limited chemostat at a dilution rate of 0.02 h^−1^ using M9 minimal medium supplemented with excess amount of sodium FA (30 mm) and continuous sparging of CO_2_‐enriched air (10 vol% of CO_2_ and 90 vol% of air). Keeping the cells under constant starvation provided a strong selection pressure to the cells and forced them to utilize CO_2_, while oxidizing FA using Fdh to produce energy source. After 350 days of chemostat culture (xylose concentration reached 0 g L^−1^ at this point), cells began to exhibit formatotrophic growth and the evolved strain isolated from the culture broth was capable of growing solely on FA and CO_2_ from an initial *OD*_600_ of 0.01 to an *OD*_600_ of 0.28 in 120 h with a doubling time of 18 ± 4 h (Table 2). To identify the mutations occurred in the genomic DNA of evolved strains, six clones were isolated at different chemostat culture time points and whole genome sequencing was performed. Among the mutation sites identified in the genome, mutations in the *prs* (encoding ribose‐phosphate diphosphokinase), *pgi* (encoding glucose 6‐phosphate isomerase), *aroH* (encoding 2‐dehydro‐3‐deoxyphosphoheptonate aldolase), and *eno* (encoding enolase) genes were speculated to have potentially contributed to formatotrophic growth by fine‐tuning of the CBB cycle flux.^[^ ^51^, ^52^, ^53^ ^]^ Further studies are needed to validate these assumptions. In addition, the maximum cell density (e.g., *OD*_600_ of 0.28; Table 2) of the evolved *E. coli* strain was too low for feasible applications in microbial chemical production.

In another study, the rTHF‐rgcv pathway and *Pseudomonas* sp. 101 Fdh were introduced in a serine‐auxotrophic *E. coli* strain, which was constructed by deleting the *ltaE* (encoding L‐threonine aldolase), *kbl* (encoding 2‐amino‐3‐ketobutyrate CoA ligase), and *aceA* (encoding isocitrate lyase) genes and replacing the native promoters of *serA* and *glyA* genes with stronger promoters in the genome, to achieve formatotrophic growth of *E. coli*.^[^ ^34^ ^]^ As a result, the engineered strain showed formatotrophic growth solely on FA and CO_2_ from an initial *OD*_600_ of 0.03 to 0.4 in 240 h with a doubling time of 70 h. To further enhance formatotrophic growth, a short‐term ALE was carried out by cultivating the cells in test tubes containing M9 minimal medium supplemented with 30 mm of sodium FA and continuous sparging of CO_2_‐enriched air (10 vol% CO_2_ and 90 vol% air). When the cell concentration reached an *OD*_600_ of 0.4, cells were transferred to a fresh culture medium to start a new cultivation (an initial *OD*_600_ of 0.03–0.05). After 13 serial cultivations (≤40 generations), the evolved strain grew up to an *OD*_600_ of 1.0 with a significantly shortened doubling time of 7.7 h (Table 2). In addition, the evolved strain showed enhanced FA to biomass conversion yield of 2.3 ± 0.2 gDCW mol FA^−1^, while the parental strain showed a yield of 1.5 gDCW mol FA^−1^. To identify the mutations occurred in the genome of the evolved strain, multiple clones exhibiting enhanced formatotrophic growth were isolated and sequenced. As a result, the 5′ untranslated region of the newly introduced *Pseudomonas* sp. (strain 101) *fdh* gene and the promoter region of *pntAB* gene encoding membrane‐bound transhydrogenase were identified to be mutated in all sequenced colonies. To confirm that the two mutant genes had contributed to the enhanced formatotrophic growth of *E. coli*, the parental *E. coli* strain was engineered to have the two mutant genes and its formatotrophic growth was compared with the parental *E. coli* strain. As a result, the parental *E. coli* strain harboring the two mutant genes showed growth characteristics (doubling time and maximum *OD*_600_) similar to the evolved strain, demonstrating that the two identified mutant genes had contributed to the enhanced formatotrophic growth of *E. coli*. Despite the great achievement of developing improved formatotrophic growth through metabolic engineering combined with ALE, the maximum cell density reached (*OD*_600_ of ≈1.0) was still too low for its use in the production of chemicals from FA and CO_2_.

### 4.2 Development of a Synthetic Formatotroph Using Rational Metabolic Engineering

Although rational metabolic engineering for the development of formatotrophic *E. coli* is more difficult compared with evolving the strain through ALE, it provides scientific basis on the key metabolic and regulatory factors required for formatotrophic growth and points direction for future studies on developing highly efficient formatotrophs. In a recent study, a synthetic formatotrophic *E. coli* strain was developed by rational metabolic engineering (Figure 1). The base strain constructed in a previous study^[^ ^31^ ^]^ by deleting the *gcvR*, *pflB*, and *serA* genes, replacing the native promoter of *gcvTHP* operon with a strong *trc* promoter, and introducing the rTHF‐rgcv pathway and *C. boidinii* Fdh (CbFdh)^[^ ^35^ ^]^ was used for further rational metabolic engineering as follows. First, the *ppsA* gene was overexpressed by replacing its native promoter with a strong *trc* promoter and the *ppsR* gene encoding PEP synthase regulator was deleted to improve cell growth on pyruvate (synthesized from FA and CO_2_) by reinforcing gluconeogenesis. Enhanced synthesis of essential metabolites, such as nucleotides and lipids, from pyruvate can be achieved by reinforcing gluconeogenesis. Second, the *purT* gene encoding phosphoribosylglycinamide formyltransferase1, which synthesizes N2‐formyl‐N1‐(5‐phospho‐*β*‐D‐ribosyl) glycinamide from N1‐(5‐phospho‐*β*‐D‐ribosyl) glycinamide and FA, was deleted as the *E. coli* strain expressing both the *purT* and *M. extorquens ftl* genes showed inferior growth compared to those expressing the *purT* and *M. extorquens ftl* genes individually.^[^ ^54^ ^]^ Third, the *Arabidopsis thaliana* Fdh mutant (mAtFdh) was introduced to resolve NADPH shortage in the cell by generating NADPH from FA; since the base strain also possesses CbFdh for generating NADH from FA, this additional engineering allows generation of both NADH and NADPH from FA. Direct NADPH generation from FA using mAtFdh can enhance formatotrophic growth as two NADPH molecules are necessary to synthesize one pyruvate molecule from FA and CO_2_. After employing the above three engineering strategies, the newly developed *E. coli* strain showed formatotrophic growth solely on FA and CO_2_ and grew up to an *OD*_600_ of 0.285 in 150 h from an initial *OD*_600_ of 0.051 with a doubling time of 60.4 h.

To further improve the formatotrophic growth of the *E. coli* strain, the overexpression levels of CbFdh and mAtFdh were optimized by employing lower copy number plasmids (copy number of 1–5) since excessive gene expression can adversely affect cell growth under nutrient‐limited culture condition (i.e., minimal medium containing FA and CO_2_ only). As a result, the formatotrophic *E. coli* strain showed improved growth solely on FA and CO_2_ and grew up to an *OD*_600_ of 0.607 in 200 h from an initial *OD*_600_ of 0.06 with a doubling time of 59.9 h. However, the cells could not grow to a higher density, which was found to be due to cell filamentation negatively affecting cell growth.^[^ ^55^ ^]^ Cell filamentation seemed to have occurred due to energy‐deficiency as cells were cultivated under nutrient‐limited culture condition.^[^ ^55^ ^]^ Hence, the energy efficiency in the cell was improved by enhancing the expression level of cytochrome *bo_3_* ubiquinol oxidase (Cyo; encoded by the *cyoABCD* gene) while decreasing the expression level of cytochrome *bd*‐I ubiquinol oxidase (Cyd; encoded by the *cydAB* and *cydX* genes) through lowering of the cultivation temperature to 32 °C. It should be noted that Cyo converts reducing powers to ATP more efficiently than Cyd as the proton translocation values of Cyo and Cyd are 2 and 1, respectively.^[^ ^56^ ^]^ Moreover, cultivating *E. coli* at lower temperatures (28 and 33 °C) were reported to increase the Cyo level and decrease the Cyd level compared with 37 °C.^[^ ^57^ ^]^ By culturing cells at 32 °C, the challenge with cell filamentation could be resolved and the engineered *E. coli* strain grew to an *OD*_600_ of 3.59 in 791.5 h from an initial *OD*_600_ of 0.018 solely on FA and CO_2_ with a doubling time of 103.6 h. Furthermore, the formatotrophic *E. coli* strain grew to an *OD*_600_ of 11.1, which is the highest cell density reported to date, in 577 h from an initial *OD*_600_ of 0.91 solely on FA and CO_2_ with a doubling time of 157.6 h in bioreactor culture (Table 2). The achievement in developing a synthetic formatotroph capable of growing to a relatively high maximum cell density presented a possibility of producing chemicals solely from FA and CO_2_. However, the lower specific growth rate is a key problem that needs to be solved.

### 4.3 Strategies to Improve Formatotrophic Growth

In order to realize an economically feasible chemical production from FA and CO_2_, a platform strain capable of growing at a much higher specific growth rate and to a higher cell density on FA and CO_2_ is needed. Thus, we suggest several engineering strategies to improve formatotrophic growth of *E. coli*. First, the gene expression levels of Cyo and Cyd at 37 °C need to be fine‐tuned in the formatotrophic strain to prevent cell filamentation by improving the energy conversion efficiency, while allowing higher growth rate compared with 32 °C. This requires a delicate engineering strategy because simply increasing the expression level of Cyo while decreasing the expression level of Cyd can lead to the formation of inclusion bodies.^[^ ^35^ ^]^ Furthermore, the *cyd* genes cannot be deleted from the genome because it is essential for cell growth.^[^ ^58^ ^]^ Such fine‐tuning of the gene expression may be performed employing for example the sRNA gene expression knockdown system,^[^ ^59^, ^60^ ^]^ which has been demonstrated to be useful for optimizing the expression levels of multiple genes.

Increased uptake and utilization of FA by the cells can also improve formatotrophic growth as the amount of energy and carbons that can be provided from one mol of FA are less than those generated from one mol of other typical carbon sources (e.g., glucose). It should be noted that one mol of FA and one mol of glucose can provide 2.5 and 32 mol of ATP through oxidative phosphorylation, respectively.^[^ ^31^ ^]^ In addition, 5.8 mol of FA is needed to synthesize one mol of pyruvate, while only 0.5 mol of glucose is required to synthesize one mol of pyruvate.^[^ ^31^ ^]^ These calculations suggest that FA needs to be transferred into the cells at least ten times faster than glucose to supply similar amount of energy and carbons through FA assimilation. The transfer of FA into the cell can be enhanced by the overexpression of genes corresponding to FA channel^[^ ^61^ ^]^ and by increasing FA concentration in the culture medium. Since the FA concentration in the medium cannot be increased to higher than ≈3 g L^−1^ because of its toxicity to the cells,^[^ ^31^ ^]^ FA tolerance of the formatotrophic *E. coli* strain needs to be enhanced by ALE^[^ ^34^ ^]^ and/or metabolic engineering.^[^ ^62^ ^]^

## 5 Opportunities for the Production of Chemicals Using the Synthetic Formatotroph

### 5.1 Metabolic Engineering Strategies for Chemical Production

**Figure 4:**
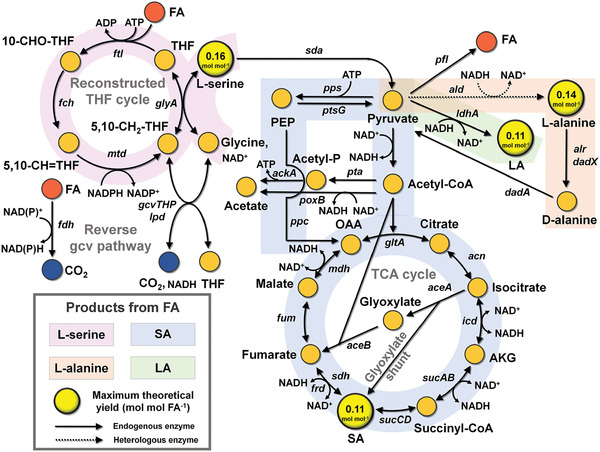
Metabolic pathways for the production of chemicals from FA and CO_2_. The metabolic pathways for the production of four chemicals (LA, L‐alanine, L‐serine, and SA), which can be produced from FA and CO_2_, using the formatotrophic *E. coli* strain employing the rTHF‐rgcv pathway are shown. The yellow circles represent the chemicals that can be produced from FA and CO_2_. The values provided in the yellow circle represent the maximum theoretical yield (mol mol FA^−1^) of the target chemical in the formatotrophic *E. coli* strain employing the rTHF‐rgcv pathway calculated by GEM simulation. Abbreviations are: SA, succinic acid; LA, lactate; 5‐10‐CH=THF, 5,10‐methenyl THF; 5,10‐CH_2_—THF, 5,10‐methlylene THF; 10‐CHO—THF, 10‐formyl THF; *aceA*, isocitrate lyase; *aceB*, malate synthase; Acetyl‐P, acetyl phosphate; *ack*, acetate kinase; *acn*, aconitate hydratase; AKG, *α*‐ketoglutarate; *ald*, alanine dehydrogenase; *alr*, alanine racemase I; *dadA*, D‐amino acid dehydrogenase; *dadX*, alanine racemase II; FA, formic acid; *fch*, 5,10‐CH=THF cyclohydrolase; *fdh*, formate dehydrogenase; *frd*, fumarate reductase; *ftl*, formate‐tetrahydrofolate ligase; *fum*, fumarase; *gcvTHP*, gcv complex; *gltA*, citrate synthase; *glyA*, serine hydroxymethyltransferase; *icd*, isocitrate dehydrogenase; *ldhA*, lactate dehydrogenase; *lpd*, lipoamide dehydrogenase; *mdh*, malate dehydrogenase; *mtd*, 5,10‐CH_2_—THF dehydrogenase; OAA oxaloacetate; PEP, phosphoenolpyruvate; *pfl*, pyruvate formate lyase; *poxB*, pyruvate dehydrogenase; *ppc*, phosphoenolpyruvate carboxylase; *pps*, phosphoenolpyruvate synthase; *pta*, phosphate acetyltransferase; *ptsG*, PTS system glucose‐specific EIICB component; *sda*, serine deaminase; *sdh*, succinate dehydrogenase; *sucAB, α*‐ketoglutarate dehydrogenase; *sucCD*, succinyl‐CoA synthetase; THF, tetrahydrofolate.

As the development of a formatotrophic *E. coli* strain capable of growing to a relatively high maximum cell density^[^ ^35^ ^]^ presented a possibility for producing chemicals solely from FA and CO_2_, we list several example chemicals, including LA, L‐alanine, L‐serine, and SA, that can be produced from FA and CO_2_. In addition, the strategies for the metabolic engineering of formatotrophic *E. coli* strain employing the rTHF‐rgcv pathway to produce these chemicals are suggested (**Figure** **4**).

LA is an important chemical used in the food and pharmaceutical industries.^[^ ^63^, ^64^ ^]^ Moreover, LA can be used as a monomer for the production of bio‐based poly(LA)^[^ ^65^, ^66^ ^]^ and its copolymers, such as poly(3‐hydroxybutyrate‐*co*‐LA).^[^ ^67^ ^]^ LA can be produced from FA and CO_2_ using formatotrophic *E. coli* strain, which produces pyruvate from FA and CO_2_, as LA can be synthesized from pyruvate through a single step reaction by lactate dehydrogenase (LdhA; Figure 4). In order to efficiently produce LA using the formatotrophic *E. coli* strain, the *ldhA* gene needs to be overexpressed and the *pta*, *ack* (encoding acetate kinase), and *pfl* genes, which convert pyruvate into other byproducts,^[^ ^68^ ^]^ can be deleted to increase the metabolic flux from FA and CO_2_ toward LA production. It should be noted that fine‐tuning of the *ldhA* gene expression is required to determine the optimal metabolic flux balance between intracellular metabolites production for cell growth and LA production from pyruvate as the formatotrophic *E. coli* strain is designed to utilize pyruvate for the synthesis of essential metabolites, including nucleotides and lipids. In addition, LA needs to be exported out of the cell rapidly to prevent or minimize LA consumption as it is a more favorable carbon source than FA.^[^ ^69^ ^]^ This can be achieved by overexpressing the *lldP* gene encoding the lactate/H^+^ symporter.^[^ ^70^ ^]^

To evaluate how efficiently FA can be converted to LA, the maximum theoretical yield of LA from FA was calculated (Figure 4) using the iML1515, a genome‐scale metabolic model (GEM) of *E. coli*.^[^ ^71^ ^]^ The rTHF‐rgcv pathway and Fdh reactions were incorporated in the iML1515 model by including three new reactions; i) FA + NAD → CO_2_ + NADH, ii) FA + NADP → CO_2_ + NADPH, and iii) glycine + NAD + THF ↔ CO_2_ + 5,10‐CH_2_—THF + NADH + NH_4_. To simulate LA production solely from FA, the reactions which uptake other carbon sources (e.g., glucose) were blocked in the GEM. The FA uptake reaction flux was set to 1 mmol gDCW^−1^ h^−1^ and the requirement for non‐growth associated maintenance energy was set to zero. The GEM simulation was carried out by setting of the maximum export metabolic flux of target chemical (LA or L‐alanine or L‐serine or SA in the cytosol is exported to the extracellular space) as an objective function under aerobic condition (no constraint on O_2_ uptake reaction). The GEM simulations were performed using cobrapy package with Gurobi Optimizer in Python 3.6 environment.^[^ ^72^ ^]^ The maximum theoretical yield of LA calculated by flux balance analysis^[^ ^73^ ^]^ is 0.11 mol of LA mol of FA^−1^ (Figure 4). In comparison, the maximum theoretical yield of LA on glucose is 1.75 mol of LA mol of glucose^−1^, which is equivalent to 0.29 mol of LA mol FA equivalent^−1^, as the number of carbons in glucose and FA differ by a factor of 6. Thus, the maximum theoretical yield of LA on glucose is 2.64 times higher than that on FA, which is due to the carbon loss during the generation of reducing power. GEM simulation revealed that 66 mol% of FA is consumed to produce reducing power, while 34 mol% of FA is consumed to produce metabolites including the target chemical.

L‐alanine, which is widely used in the food, pharmaceutical, and veterinary industries,^[^ ^74^, ^75^ ^]^ is another chemical that can be produced from FA and CO_2_ using a formatotrophic *E. coli* strain. In addition, L‐alanine is utilized to synthesize various polymers, such as co‐polyamides.^[^ ^76^, ^77^ ^]^ L‐alanine can be produced from pyruvate through a single step reaction by alanine dehydrogenase (Ald; Figure 4). In order to efficiently produce L‐alanine using the formatotrophic *E. coli* strain, the *pta*, *ack*, *ldhA*, and *pfl* genes need to be deleted to increase the metabolic flux toward L‐alanine formation by preventing pyruvate from being converted to various byproducts.^[^ ^78^ ^]^ Moreover, the *E. coli* Ald, which converts pyruvate to L‐alanine by consuming intracellular metabolites such as L‐glutamate or L‐valine,^[^ ^78^ ^]^ needs to be replaced with the heterologous Ald (e.g., *Geobacillus stearothermophilus* Ald), which converts pyruvate to L‐alanine by consuming NADH, to achieve both formatotrophic growth and L‐alanine production.^[^ ^78^ ^]^ Furthermore, the *dadA* gene encoding D‐amino acid dehydrogenase needs to be deleted to prevent L‐alanine consumption as alanine racemase I and II encoded by the *alr* and *dadX* genes, respectively, convert L‐alanine to D‐alanine, which is further converted to pyruvate by the D‐amino acid dehydrogenase.^[^ ^79^ ^]^ The maximum theoretical yield of L‐alanine on FA by GEM simulation is 0.14 mol of L‐alanine mol of FA^−1^ (Figure 4).

L‐serine, which is also widely used in the food, cosmetics, and pharmaceutical industries,^[^ ^80^, ^81^, ^82^ ^]^ is another *α*‐amino acid that can be directly synthesized through the rTHF‐rgcv pathway in the formatotrophic *E. coli* strain (Figure 4). In order to efficiently produce L‐serine using the formatotrophic *E. coli* strain, fine‐tuning of the expression level of the *sda* gene using metabolic engineering strategies, such as sRNA^[^ ^59^ ^]^ or CRISPRi,^[^ ^83^ ^]^ is necessary since Sda is an important enzyme influencing both formatotrophic growth and L‐serine production. Moreover, minimizing the reverse reaction activity of the bidirectional GlyA, which degrades L‐serine into glycine and 5,10‐CH_2_—THF (Figure 4), can be beneficial for enhanced L‐serine production. The reverse reaction activity of GlyA can be reduced by enzyme evolution or replacement of native enzyme with a heterologous enzyme possessing higher forward reaction activity and lower reverse reaction activity.^[^ ^84^ ^]^ Overexpression of the *eamA* gene encoding the cysteine/homoserine transporter would be beneficial to reduce L‐serine degradation by facilitating L‐serine export to the culture medium.^[^ ^85^ ^]^ The maximum theoretical yield of L‐serine on FA is 0.16 mol of L‐serine mol of FA^−1^ (Figure 4).

SA is recognized as one of the most useful bio‐based chemicals due to its use as a precursor for numerous industrially valuable chemicals^[^ ^37^, ^86^ ^]^ and as a monomer for the synthesis of various bio‐based polymers, including polyesters and polyamides (Nylon x,4).^[^ ^87^ ^]^ Unlike LA, L‐alanine, and L‐serine, which are directly produced from pyruvate or the rTHF‐rgcv pathway, a series of reactions from pyruvate are required to produce SA in the formatotrophic *E. coli* strain. In order to efficiently produce SA using the formatotrophic *E. coli* strain, metabolic engineering of the formatotrophic *E. coli* strain needs to be performed for aerobic SA production. As the formatotrophic *E. coli* strain is cultivated under nutrient‐limited culture condition, O_2_ supply through aeration is essential to produce ATP from reducing powers. Moreover, reducing powers are generated through the oxidative tricarboxylic acid (TCA) cycle, offering advantages in terms of rapid cell growth and high SA productivity. However, SA cannot be produced in the formatotrophic *E. coli* strain under aerobic condition due to the presence of succinate dehydrogenase (encoded by the *sdhAB* gene), which converts SA to fumarate. Thus, the *sdhAB* gene needs to be deleted to enable SA accumulation in the formatotrophic *E. coli* strain.^[^ ^88^ ^]^ In addition, utilization of oxidative TCA cycle together with the glyoxylate shunt pathway can offer higher SA yield as the glyoxylate shunt pathway does not involve oxidative decarboxylation.^[^ ^86^ ^]^ Hence, the *iclR* gene, encoding the transcriptional repressor for the glyoxylate shunt pathway, needs to be deleted to activate this pathway. SA production from FA and CO_2_ can be further improved by deleting the *poxB* (encoding pyruvate dehydrogenase), *pta*, *ack*, *and ldhA* genes, which convert pyruvate to other byproducts,^[^ ^88^ ^]^ and by increasing the expression level of Fdh, which was demonstrated to increase SA production through enhanced NADH supply.^[^ ^89^, ^90^ ^]^ The maximum theoretical yield of SA on FA is 0.11 mol of SA mol of FA^−1^ (Figure 4).

The maximum theoretical yields of bio‐derived chemicals (including the above example chemicals) on FA are lower than those on glucose due to several reasons including the use of FA to generate reducing powers. This lower maximum theoretical yield can be compensated by the lower cost of FA ($200 ton^−1^) compared with glucose ($300–400 ton^−1^) and CO_2_ utilization contributing to “net zero” vision globally set by using FA derived from CO_2_.^[^ ^23^ ^]^ Since fossil resource‐based production of chemicals is expected to be associated with penalty costs, it is increasingly important to reduce CO_2_ generation and utilize CO_2_ for chemical production. Thus, in addition to the lignocellulosics‐based biorefineries currently pursued actively, bio‐based chemical production by formatotrophic microorganisms using FA (derived from CO_2_) and CO_2_ as sole carbon sources will be of great importance. To make the one‐carbon biorefinery more economically competitive, other systems that allow supply of reducing powers can be integrated; for example, renewable electricity can be supplied to generate NADH/NADPH, while a majority of FA is used to grow cells and produce desired chemicals.

### 5.2 Development of Fermentation Processes

Besides the development of a formatotrophic *E. coli* strain to produce chemicals, establishment of an optimal fermentation process is equally important for enhancing formatotrophic growth and production indices (titer, yield, and productivity) of a target chemical. There are a number of factors that need to be optimized, including culture medium, temperature, pH (and pH controlling agent), aeration, and nutrient feeding strategy (including FA supplementation). Among them, let us examine three factors, aeration, pH controlling agents, and FA feeding strategy, as examples of how these factors need to be considered.

Aeration determines the growth of the formatotrophic *E. coli* strain, as reducing powers are converted to ATP through oxidative phosphorylation.^[^ ^35^ ^]^ Thus, increasing aeration can enhance oxidative phosphorylation, resulting in higher cell growth. To increase aeration, the dissolved O_2_ level in the culture broth, which is measured to control the aeration in a bioreactor by regulating the rotor speed, needs to be set higher than that of a normal *E. coli* fermentation. Potential cell damage caused by higher shear stress under these conditions needs to be examined as well. Moreover, increasing air flow rate (or use of pure oxygen) and changing the type of impeller^[^ ^91^

^]^ or sparger^[^ ^92^ ^]^ can be feasible options for enhancing aeration and to achieve higher formatotrophic growth.^[^ ^93^ ^]^

Selection of the best pH controlling agent is another important factor to develop an optimal fermentation process for the formatotrophic *E. coli* strain as hydroxide is generated from the consumption of FA ions and increases the pH of the fermentation broth. Thus, supplementation of an acid (e.g., HCl) is required to neutralize the pH of fermentation broth. However, the addition of an acid can be detrimental to cell growth due to the accumulation of toxic compounds (e.g., KCl).^[^ ^94^ ^]^ Pure FA can be the most feasible option for controlling the pH of the fermentation since no deleterious compounds are generated from FA. Moreover, controlling of pH and replenishing of FA (as a carbon source) in the bioreactor can be carried out simultaneously by selecting FA as a pH controlling agent. Such “dual purpose” strategy of using FA can simplify the entire bioprocess for chemical production using FA and CO_2_.

Designing an optimal FA feeding strategy is probably the most important for developing an optimal process for the fermentation of a formatotrophic *E. coli* strain. FA in the culture broth at medium to high concentrations (tens of mm)^[^ ^35^, ^37^ ^]^ negatively affects formatotrophic growth by reducing ATP production in the cell as FA inhibits cytochrome c oxidase, which is an enzyme associated with the respiratory electron transport.^[^ ^95^ ^]^ Moreover, the cytoplasm acidifies due to the diffusion of protonated FA across the cell membrane and reduces the proton motive force.^[^ ^38^, ^95^ ^]^ Furthermore, FA concentration above ≈3 g L^−1^ in the culture broth was found to inhibit growth of *E. coli* (and some other bacteria such as *Mannheimia succiniciproducens*) in previous studies.^[^ ^35^, ^37^ ^]^ Thus, a fed‐batch fermentation process equipped with an automated FA feeding controller needs to be well established to maintain the FA concentration and pH in the culture broth within the optimal range. As mentioned earlier, it will be desirable to increase the FA tolerance of the formatotrophic strain through additional metabolic engineering. This will allow higher FA consumption rate and ultimately lead to higher growth rate and product formation rate.

## 6 Conclusions

As the world is moving toward “net zero” vision, bio‐based production of fuels, chemicals, and materials from renewable resources is becoming increasingly important. Also, the use of one carbon chemicals such as CO_2_, CO, and CH_4_ as substrates for chemical production (thus, one‐carbon biorefinery) has recently been attracting attention. In this paper, we reviewed recent advances in the construction of synthetic FA and CO_2_ assimilation pathways and the development of formatotrophic *E. coli* strain. After summarizing current achievements, we raised current challenges for establishing one‐carbon biorefinery together with possible solutions. In addition, we analyzed the possibility of producing several chemicals, together with the strategies for metabolic engineering of the formatotrophic *E. coli* strain to produce these chemicals. Finally, several factors to be considered to optimize fermentation for efficiently producing chemicals from FA and CO_2_ were discussed.

In addition to developing an efficient formatotrophic platform strain, it is necessary to establish an optimal FA production system (e.g., electrochemical reduction,^[^ ^20^ ^]^ photoreduction,^[^ ^96^ ^]^ and hydrogenation of CO_2_^[^ ^97^ ^]^), which enables economically competitive production of chemicals from FA and CO_2_ when coupled with the bioprocess (i.e., fermentative production using a metabolically engineered formatotroph). Current technologies already show satisfactory efficiencies in electrochemical reduction of CO_2_ to FA (Faradaic efficiency above 90%), and thus significantly reducing the production cost of FA from CO_2_.^[^ ^21^, ^23^ ^]^ On the other hand, electrochemical reduction of CO_2_ to two (e.g., ethanol) or three (e.g., propanol) carbon chemicals is inefficient (Faradaic efficiency ≈50%) mainly due to low conversion efficiency and byproduct formation.^[^ ^98^ ^]^ Moreover, only a few chemicals can be produced from electrochemical reduction of CO_2_ using different kinds of electrocatalysts or changing the electrode potentials,^[^ ^99^ ^]^ while many different chemicals can be produced from FA by the formatotrophic microorganisms using the metabolic pathways designed and established for the production of those chemicals.^[^ ^100^ ^]^ Therefore, the electrochemical reduction of CO_2_ to FA followed by FA conversion to other chemicals using the formatotrophic microorganisms is much more advantageous. Studies on integrating electrochemical and fermentation systems for diverse applications are being actively pursued^[^ ^101^ ^]^ and various configurations of the integrated system have been explored for efficient electricity‐dependent microbial fermentation.^[^ ^102^, ^103^ ^]^ With such effort, development of optimal electrochemical–biological hybrid systems for the efficient production of chemicals from FA and CO_2_ is expected in the near future. When these challenges are resolved, it is expected that various chemicals^[^ ^100^ ^]^ can be produced from FA and CO_2_. We hope that this review will provide guidance and considerations for establishing a sustainable and economical one‐carbon biorefinery for the production of chemicals from FA and CO_2_.

## Conflict of Interest

The authors declare no conflict of interest.
